# Super-resolution deep learning reconstruction improves the depiction of peripancreatic arteries and image quality in pancreatic cancer CT

**DOI:** 10.1007/s00261-026-05596-w

**Published:** 2026-06-13

**Authors:** Rin Tsujimoto, Koichiro Yasaka, Takatoshi Kubo, Satoru Kamio, Akira Katayama, Yusuke Asari, Ako Negishi, Osamu Abe

**Affiliations:** The University of Tokyo, Tokyo, Japan

**Keywords:** Pancreatic neoplasms, Arteries, Tomography, X-ray computed, Deep learning, Image processing, Computer-assisted

## Abstract

**Purpose:**

Accurate identification of peripancreatic arteries in pancreatic ductal adenocarcinoma (PDAC) is essential for determining surgical strategy and prognosis. This study compared super-resolution deep learning reconstruction (SR-DLR) with normal-resolution deep learning reconstruction (NR-DLR) and hybrid iterative reconstruction (HIR) in the depiction of peripancreatic arteries in patients with PDAC undergoing pancreatic dynamic CT.

**Methods:**

This retrospective study included 40 patients (mean age, 70.8 ± 9.3 years) with pathologically confirmed PDAC who underwent pancreatic dynamic CT. Images were reconstructed using SR-DLR, NR-DLR, and HIR. In quantitative image analyses, CT attenuation, image noise, contrast-to-noise ratio (CNR), edge rise slope (ERS), and edge rise distance (ERD) were assessed. In qualitative image analyses, five radiologists independently evaluated arterial depiction of major arteries and small peripancreatic arteries, tumor–vessel interface, image noise, image sharpness, diagnostic acceptability, and tumor conspicuity. Statistical analyses were performed using paired *t*-tests and the Wilcoxon signed-rank test with Bonferroni correction.

**Results:**

SR-DLR showed higher CT attenuation, lower image noise, and higher CNR than NR-DLR and HIR (all *p* < 0.001). For both the superior mesenteric artery and pancreatic parenchyma, ERS was higher with SR-DLR than with NR-DLR and HIR (all *p* ≤ 0.002), whereas ERD was lower than with NR-DLR and HIR (all *p* ≤ 0.001). In qualitative image analyses, SR-DLR achieved the highest scores for small peripancreatic arteries and all qualitative items except for the depiction of major peripancreatic arteries (*p* < 0.001).

**Conclusion:**

SR-DLR improved quantitative image quality and enhanced the qualitative depiction of small peripancreatic arteries and the tumor–vessel interface in pancreatic dynamic CT for patients with PDAC.

**Graphical Abstract:**

**Figure:**
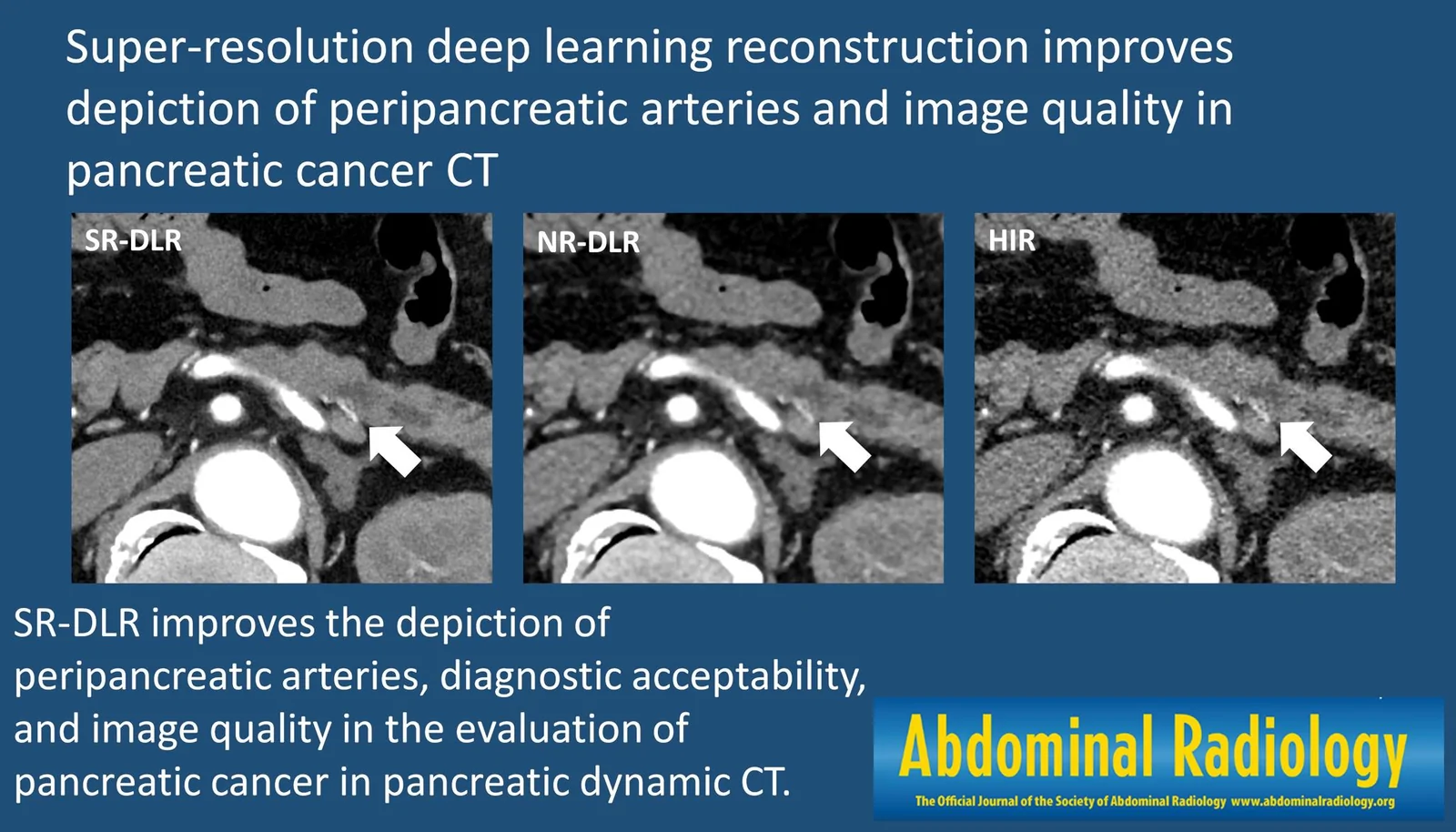

## Introduction

Pancreatic ductal adenocarcinoma (PDAC) remains one of the most lethal malignancies, and contrast-enhanced multiphase pancreatic CT plays a central role in its detection, staging, and assessment of resectability [1]. In particular, accurate evaluation of the relationship between the tumor and surrounding vessels is crucial for determining surgical strategy and prognosis [2].

According to the National Comprehensive Cancer Network guidelines (version 2.2026), assessment of tumor contact with major arteries—including the celiac artery, common hepatic artery, proper hepatic artery, and superior mesenteric artery—is essential for determining resectability [3]. However, in clinical practice, a more detailed understanding is required, including the gastroduodenal artery, pancreaticoduodenal arcade system, dorsal pancreatic artery, and the root of the splenic artery [4]. For example, in pancreaticoduodenectomy, ligation of the gastroduodenal artery and the inferior pancreaticoduodenal artery at their origins is necessary for vascular control, and knowledge of their branching patterns and the distance between the tumor and the arteries is critical [5]. In recent years, the artery-first approach has been increasingly adopted; early division of the inferior pancreaticoduodenal artery can reduce intraoperative blood loss by decreasing blood flow to the pancreatic head [6, 7]. In distal pancreatectomy, evaluation of the distance between the tumor and the root of the splenic artery is also important [8, 9]. Furthermore, pancreatic body and tail cancers may extend along the dorsal pancreatic artery, requiring not only division at its origin but also dissection of the surrounding soft tissue along the artery [10]. These peripancreatic arteries exhibit substantial anatomical variations, and detailed preoperative imaging assessment is essential to avoid unexpected intraoperative bleeding [11].

Accurate evaluation of these vessels requires high spatial resolution capable of depicting peripancreatic arteries, as well as clear delineation of the tumor–vessel interface. However, increasing spatial resolution in CT generally leads to increased image noise, which may reduce diagnostic performance and confidence, particularly in the evaluation of small vascular structures [12, 13]. Recently, deep learning reconstruction (DLR) has been introduced to overcome this trade-off by reducing image noise while preserving image texture [14–16]. Most currently used DLR techniques are normal-resolution DLR (NR-DLR), which reduce image noise while maintaining a more favorable noise texture compared with hybrid iterative reconstruction (HIR). Furthermore, super-resolution DLR (SR-DLR), which enables high-resolution reconstruction such as a 1024 × 1024 matrix, has been reported to improve spatial resolution and vascular visualization in abdominal CT [17].

Previous studies have demonstrated that SR-DLR improves image quality and diagnostic confidence in the evaluation of PDAC, pancreatic parenchyma, and major vessels [18]. However, few studies have focused on peripancreatic arteries, such as the pancreaticoduodenal arcade system and the dorsal pancreatic artery–transverse pancreatic artery–great pancreatic artery system, and investigated the impact of reconstruction methods on their visualization and the assessment of tumor–vessel relationships.

The aim of this study was to evaluate whether SR-DLR, as the reconstruction method of primary interest, improves the visualization of peripancreatic arteries and image quality compared with NR-DLR and HIR.

## Materials and methods

Our Institutional Review Board approved this retrospective study, and the requirement for obtaining written informed consent was waived.

### Patients

**Fig. 1:**
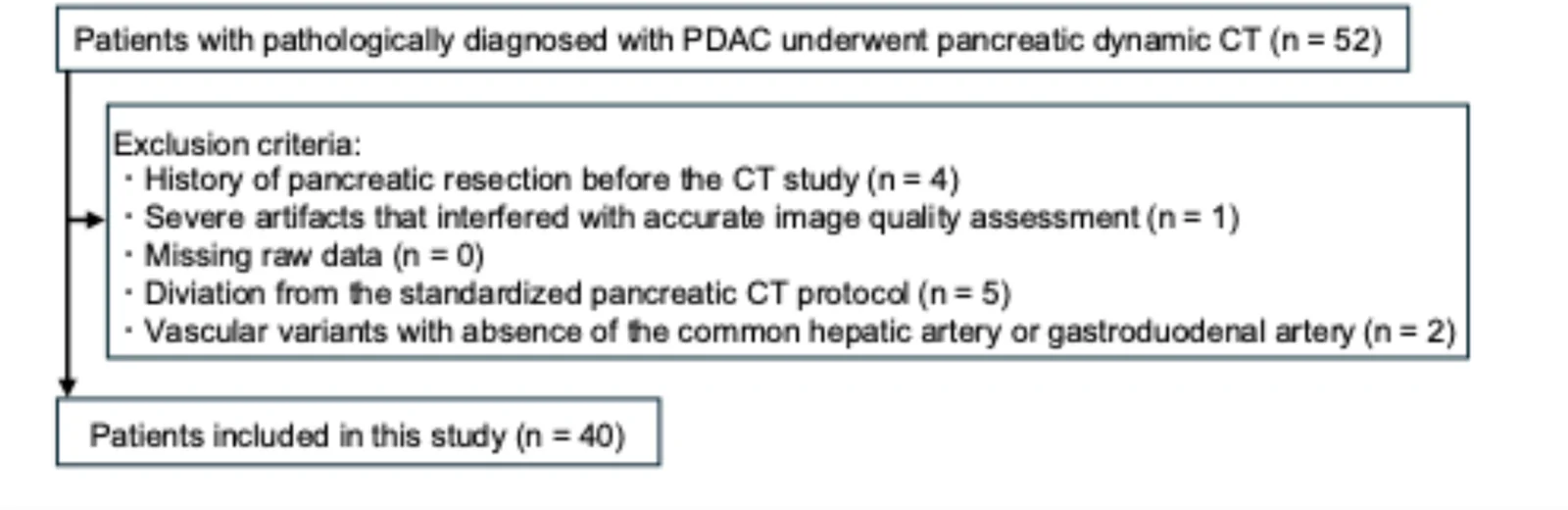
Flowchart of patient inclusion process. *PDAC* pancreatic ductal adenocarcinoma

We retrospectively identified patients who underwent pancreatic dynamic CT between March 2025 and August 2025 and were pathologically diagnosed with PDAC. A total of 52 patients met the inclusion criteria. Patients were excluded if they had a history of pancreatic resection before the CT study, exhibited severe artifacts that interfered with accurate image quality assessment, lacked the raw data necessary for retrospective image reconstruction, underwent CT with contrast protocols different from the standardized pancreatic CT protocol, or had vascular variants with absence of the common hepatic artery or gastroduodenal artery. After applying these exclusion criteria, our study included 40 patients (25 males and 15 females; mean age: 70.8 ± 9.3 years). Figure 1 summarizes the patient inclusion process. Table 1 shows age, sex, body mass index, CT dose index volume, dose length product, tumor maximum diameter, tumor location, presence of metastasis, and resectability status.

## CT imaging and reconstruction

All patients underwent CT examination including the abdominal region with a single scanner (Aquilion ONE INSIGHT Edition [Canon Medical Systems, Otawara, Japan]). CT imaging was performed using the following parameters: tube voltage, 120 kVp; automatic tube current modulation with a standard deviation of 13.0, defined for a reference slice thickness of 5 mm; helical scanning; beam pitch, 0.813; and gantry rotation time, 0.5 s. Contrast material with an iodine dose of 600 mgI/kg was administered intravenously via the antecubital vein over 30 s using a power injector. Early arterial phase and late arterial phase images were acquired 25 s and 45 s after the start of contrast injection, respectively. Radiation dose indices, including CT dose index volume (CTDIvol) and dose-length product (DLP), were recorded for each arterial phase. Because identical scanning parameters were used for the early and late arterial phases, CTDIvol was identical between the two phases in all patients.

CT images were reconstructed with the following three algorithms developed by Canon Medical Systems:

・Precise IQ Engine (SR-DLR).

・Advanced intelligent Clear-IQ Engine (NR-DLR).

・Adaptive iterative dose reduction three-dimensional (HIR).

Precise IQ Engine is a second-generation super-resolution algorithm developed to improve spatial resolution while maintaining signal fidelity under routine clinical scanning conditions, whereas Advanced Intelligent Clear-IQ Engine is a first-generation deep learning reconstruction algorithm primarily focused on noise reduction. Additionally, a conventional hybrid iterative reconstruction algorithm, Adaptive iterative dose reduction three-dimensional, was employed, which has been widely adopted in Canon CT systems to achieve noise reduction compared with filtered back projection while preserving clinically acceptable reconstruction times.

For image analyses, all images were reconstructed with a slice thickness and increment of 1 mm. The field of view was set to adequately include the entire pancreas and was kept consistent across the three reconstruction algorithms. The z-axis scan range extended from the upper margin of the liver to the lower margin of the kidneys. The matrix size of SR-DLR was 1024 × 1024, whereas that of NR-DLR and HIR was 512 × 512. These matrix sizes were maximum settings for each reconstruction algorithm. Reconstruction strength level of 2, which corresponds to “Standard” setting in previous Canon CT systems, was used for SR-DLR, NR-DLR, and HIR.

## Quantitative image analyses

**Fig. 2:**
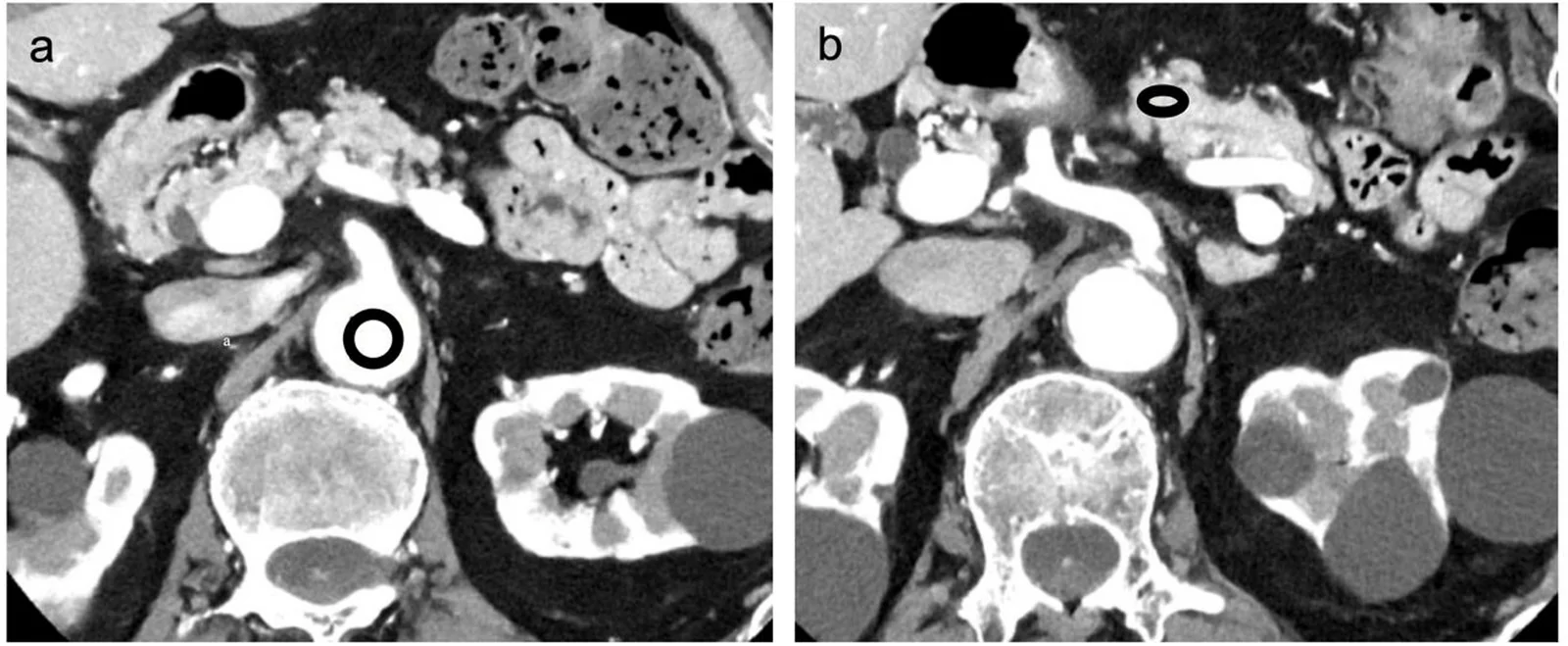
Circular or ovoid regions of interest were placed on **a** the aorta (black circle) and **b** pancreatic parenchyma (black circle)

Quantitative image analyses were conducted using ImageJ software (http://imagej.nih.gov/ij). A radiologist with 3 years of experience in diagnostic imaging (Radiologist A), under the supervision of a senior radiologist with 16 years of experience (Radiologist B), placed ovoid regions of interest (ROIs) on the abdominal aorta at the slice level corresponding to the origin of the superior mesenteric artery on early arterial phase and late arterial phase images. In addition, on late arterial phase images, ovoid ROIs were placed on the pancreatic parenchyma (Fig. 2).

The mean CT attenuation and standard deviation within each ROI were measured. The ROI area for the abdominal aorta was approximately 85 mm², with care taken to avoid the vessel wall and atherosclerotic plaques. The ROI area for the pancreatic parenchyma was approximately 45 mm² and was carefully positioned to avoid visible vessels, the pancreatic duct, focal lesions, and image artifacts. ROIs were initially placed on the SR-DLR images, and the same ROIs were subsequently copied to the NR-DLR and HIR images to ensure identical ROI location, size, and shape across the different reconstruction algorithms. To minimize measurement bias, ROIs were placed without referring to the measured CT attenuation or SD values; these values were recorded only after ROI placement was finalized. Image noise was defined as the standard deviation (SD) of CT attenuation within the abdominal aortic ROI. The SD measured on early arterial phase and late arterial phase images was defined as SD_aorta1_ and SD_aorta2_, respectively. The contrast-to-noise ratio (CNR) was calculated as follows: *CNR = (CT*_*aorta2*_ - *CT*_*pancreas*_*) / SD*_*aorta2*_, where CT_aorta2_ and CT_pancreas_ represent the mean CT attenuation values of the abdominal aorta and pancreatic parenchyma measured on late arterial phase images, respectively.

For quantitative evaluation using area-based ROIs, the abdominal aorta was used as a surrogate vascular structure rather than pancreatic feeding vessels. Because pancreatic feeding vessels are small in caliber, direct ROI placement within the vessel lumen is susceptible to partial volume effects and limited reproducibility. Therefore, direct measurements within pancreatic feeding vessels were avoided, and the abdominal aorta was selected to enable stable and robust quantitative comparisons across reconstruction algorithms.

**Fig. 3:**
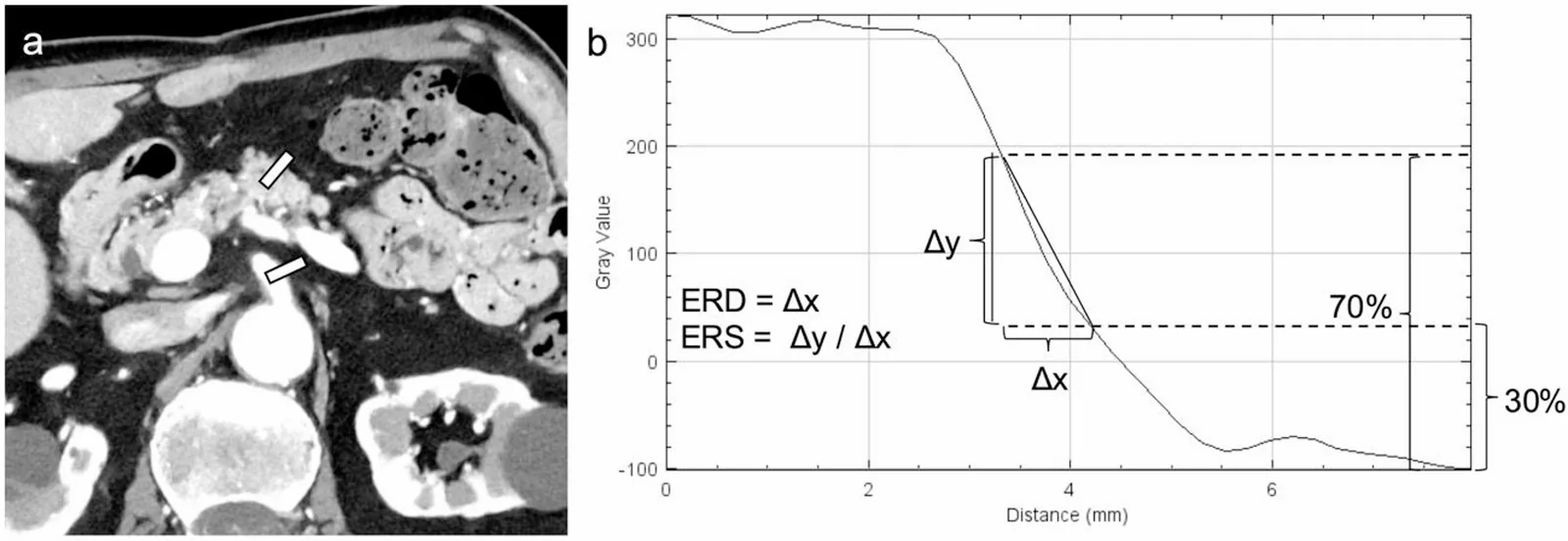
A linear region of interest (white line) was placed transversely across the interface between the pancreatic parenchyma and adjacent fat, and the wall of the superior mesenteric artery (**a**). From the CT attenuation profile along the linear region of interest, edge rise distance (ERD) and edge rise slope (ERS) were calculated with setting the reference points on the curve with 30%–70% values (**b**)

Linear ROIs were placed perpendicular to the interfaces between the pancreatic parenchyma and adjacent fat, as well as between the superior mesenteric artery and surrounding fat, on the SR-DLR images in the late arterial phase (Fig. 3) and were copied to the corresponding NR-DLR and HIR images. These linear ROIs were carefully positioned to avoid visible artifacts and calcifications. From the CT attenuation profiles obtained along the linear ROIs, the edge rise distance (ERD) and edge rise slope (ERS) were calculated (Fig. 3). ERD and ERS were calculated from the CT attenuation profiles using the 30%–70% reference points of the attenuation transition. Percentage-based thresholds on line density or CT attenuation profiles have been used in previous studies to quantify edge sharpness, including 10%–90% and 20%–80% ranges [18, 19]. In the present study, the 30%–70% range was selected to focus on the central portion of the attenuation transition because the pancreatic parenchyma–fat interface has a relatively smaller attenuation difference than high-contrast vascular interfaces and may be more susceptible to noise and local heterogeneity at the peripheral portions of the profile. The same threshold definition was applied consistently to all reconstruction algorithms.

## Qualitative image analyses

Qualitative image analyses were performed by five radiologists (readers 1–5) with 13, 7, 5, 4, and 2 years of experience in diagnostic radiology, respectively. Prior to image interpretation, all image datasets were anonymized and randomized by an independent radiologist. The readers were blinded to patient clinical information and reconstruction methods. All images were reviewed on a single workstation using ImageJ software. Images were displayed at the same size and were evaluated using predefined scoring criteria.

The qualitative image analyses included the following evaluation items:

- Vascular depiction of major peripancreatic arteries, including the celiac artery, common hepatic artery, proper hepatic artery, superior mesenteric artery, splenic artery, and gastroduodenal artery (4 = clear depiction, 3 = unclear depiction in some regions, 2 = moderately unclear depiction with difficulty in assessing vessel continuity in certain segments, 1 = poor depiction with difficulty identifying the vascular course).
- Vascular depiction of small peripancreatic arteries, including the pancreaticoduodenal arcade system and the dorsal pancreatic artery–transverse pancreatic artery–great pancreatic artery system (4 = clear depiction, 3 = unclear depiction in some regions, 2 = moderately unclear depiction with difficulty in assessing vessel continuity in certain segments, 1 = poor depiction with difficulty identifying the vascular course).
- Assessment of the ease of evaluating tumor–vessel interface for vascular invasion (4 = clear tumor–vessel interface, allowing confident assessment of the presence or absence of vascular invasion, 3 = partially unclear tumor–vessel interface, 2 = moderately unclear tumor–vessel interface with residual uncertainty regarding vascular contact or invasion, 1 = indistinguishable tumor–vessel interface, precluding assessment of vascular invasion).
- Image noise (4 = no or minimal noise, 3 = mild noise, 2 = moderate noise, 1 = severe noise interfering with diagnosis).
- Image sharpness (4 = sharp structural margins, 3 = slightly blurred margins in some regions, 2 = moderately blurred margins, 1 = blurred margins in most regions).
- Diagnostic acceptability for evaluating arterial anatomy and vascular invasion (5 = excellent image quality sufficient for diagnosis, 4 = slightly better than standard image quality, 3 = standard image quality with acceptable diagnostic confidence, 2 = somewhat unsuitable for diagnosis, 1 = unacceptable for diagnosis). In this scale, “standard image quality” for a score of 3 referred to the minimum level of image quality considered diagnostically acceptable for routine interpretation. This evaluation item was influenced by multiple factors, including image noise, sharpness, and vascular depiction.
- Tumor conspicuity of PDAC (4 = clearly depicted tumor with minimal image noise, 3 = slightly blurred tumor margin with mild image noise, 2 = unclear but identifiable tumor with noticeable image noise, 1 = tumor presence barely identifiable).

The visual evaluation was performed using standard abdominal window settings. To assess the clinical relevance of score improvements, we additionally calculated the proportion of cases in which the per-patient median score improved with SR-DLR. Score improvement was defined as an increase of at least one point with SR-DLR compared with NR-DLR or HIR. For the evaluation of small peripancreatic arteries, diagnostic conversion was defined as an improvement from a diagnostically insufficient score (score ≤ 2), indicating difficulty in assessing vessel continuity in certain segments, to a diagnostic score (score ≥ 3) with SR-DLR.

## Statistical analyses

Statistical analyses were performed using EZR version 1.70 (https://www.jichi.ac.jp/usr/hema/EZR/statmed.html). Quantitative values and qualitative scores were compared for SR-DLR vs. NR-DLR and SR-DLR vs. HIR using the paired *t* test and the Wilcoxon signed-rank test, respectively. In the qualitative image analyses, the median score of the five readers was calculated for each patient, and these per-patient median scores were used for all statistical analyses. Due to multiple comparisons (SR-DLR vs. NR-DLR and SR-DLR vs. HIR), Bonferroni correction was applied, and a *p* value of < 0.025 (= 0.050/2) was considered to indicate a statistically significant difference. Inter-reader agreement among the five radiologists was assessed for each qualitative assessment item using intraclass correlation coefficients (ICCs) across all reconstructed image sets. ICC(2,1), based on a two-way random-effects model for absolute agreement and single measurements, was used. ICC values were interpreted as follows: < 0.40, poor agreement; 0.40–0.75, fair to good agreement; and 0.76–1.00, excellent agreement.

**Table 1:** Patient characteristics

	Number or mean ± SD
Age (years)	70.8 ± 9.3
Sex (male, female)	25, 15
Body mass index (kg/m^2^)	21.5 ± 3.3
CT dose index volume (mGy)	14.9 ± 1.6
Dose length product (mGy・cm)	433.6 ± 74.1
Tumor maximum diameter (mm)	33.8 ± 19.1
Tumor location (pancreatic head, body, tail)	18, 9, 13
Presence of metastasis	22/40
Resectability status (R, BR, UR)	11, 2, 27

*SD* standard deviation, *R* resectable, *BR* borderline resectable, *UR* unresectable

## Results

### Quantitative image analyses

The results for the quantitative image evaluation are summarized in Table 2. CT attenuation of the aorta was significantly higher with SR-DLR than with NR-DLR and HIR in both the early and late arterial phases, and CT attenuation of the pancreatic parenchyma on late arterial phase images was also significantly higher with SR-DLR than with NR-DLR and HIR (all *p* < 0.001). Image noise was significantly lower with SR-DLR than with NR-DLR and HIR (all *p* < 0.001). Similarly, CNR of the pancreatic parenchyma was significantly higher with SR-DLR than with NR-DLR and HIR (all *p* < 0.001).

Regarding edge sharpness, ERS values of the superior mesenteric artery were significantly higher with SR-DLR than with NR-DLR (*p* = 0.002) and HIR (*p* < 0.001). ERS values of the pancreatic parenchyma were also significantly higher with SR-DLR than with NR-DLR and HIR (all *p* < 0.001). ERD of the pancreatic parenchyma was significantly lower with SR-DLR than with NR-DLR (*p* = 0.001) and HIR (*p* < 0.001). Similarly, ERD of the superior mesenteric artery was significantly lower with SR-DLR than with NR-DLR and HIR (all *p* < 0.001).

## Qualitative image analyses

**Fig. 4:**
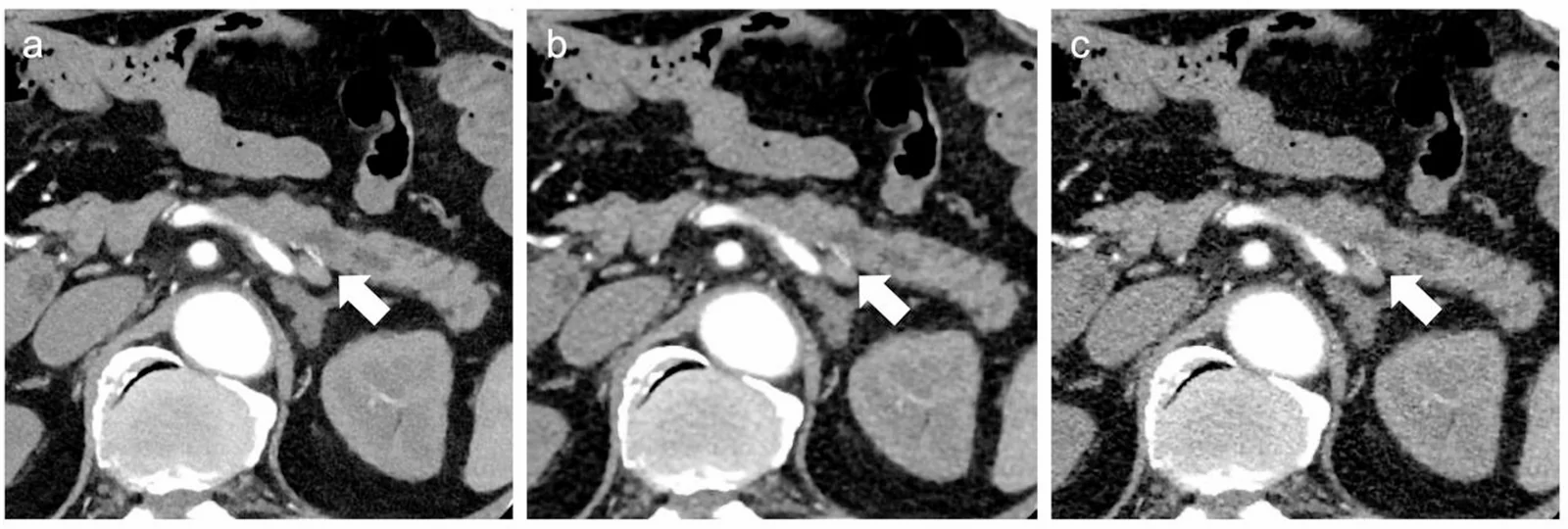
CT images of a 77-year-old male patient reconstructed using super-resolution deep learning reconstruction (SR-DLR) (**a**), normal-resolution deep learning reconstruction (NR-DLR) (**b**), and hybrid iterative reconstruction (HIR) (**c**). The dorsal pancreatic artery–transverse pancreatic artery–great pancreatic artery system (arrows) is more sharply delineated in SR-DLR than with NR-DLR and HIR, without increased image noise

**Fig. 5:**
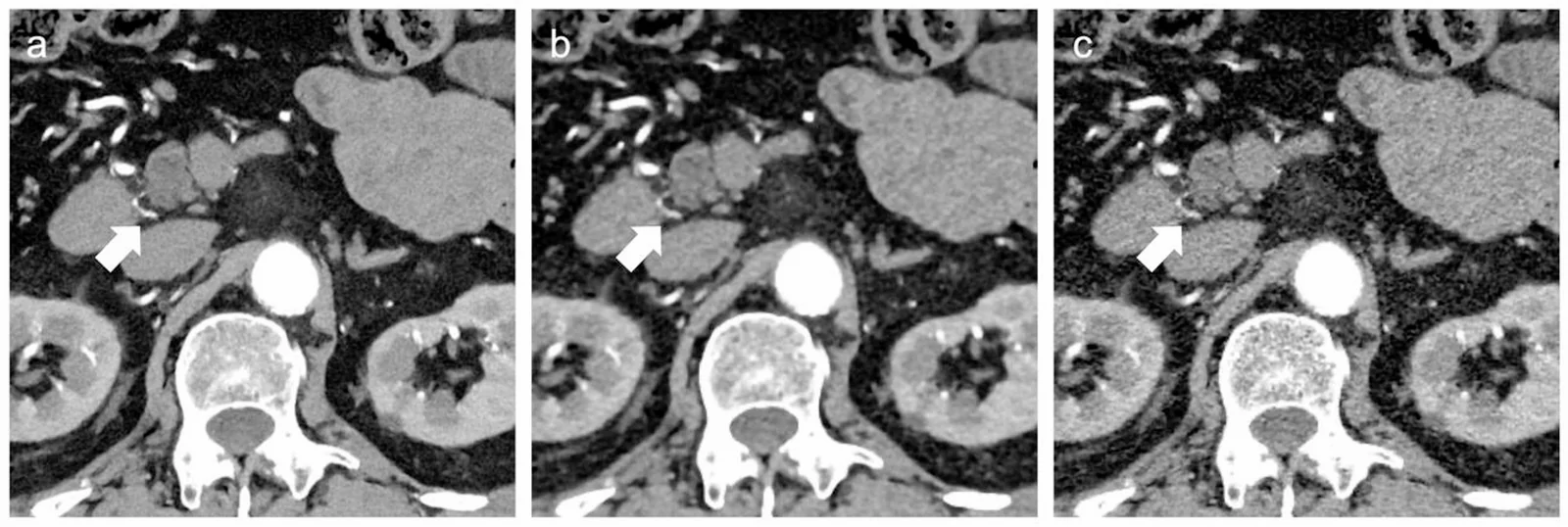
CT images of a 60-year-old male patient reconstructed using super-resolution deep learning reconstruction (SR-DLR) (**a**), normal-resolution deep learning reconstruction (NR-DLR) (**b**), and hybrid iterative reconstruction (HIR) (**c**). The pancreaticoduodenal arcade system (arrows) is more clearly delineated in SR-DLR than with NR-DLR and HIR, without increased image noise

The results for the qualitative image evaluation are summarized in Table 3. For the major arteries, including the celiac artery, common hepatic artery, proper hepatic artery, superior mesenteric artery, splenic artery, and gastroduodenal artery, all case-level median scores were identical between SR-DLR and both NR-DLR and HIR; therefore, the Wilcoxon signed-rank test was not applicable for these comparisons. In contrast, SR-DLR yielded higher scores than both NR-DLR and HIR for small peripancreatic arteries, including the pancreaticoduodenal arcade system and the dorsal pancreatic artery–transverse pancreatic artery–great pancreatic artery system (all *p* < 0.001) (Figs. 4 and 5). For the pancreaticoduodenal arcade system, SR-DLR improved the score from 3 to 4 in 27 of 40 cases (67.5%) compared with NR-DLR and in 33 of 40 cases (82.5%) compared with HIR. However, because all cases were scored within the diagnostic range across all reconstruction methods, no diagnostic conversion was observed. For the dorsal pancreatic artery–transverse pancreatic artery–great pancreatic artery system, score improvement with SR-DLR was observed in 16 of 40 cases (40.0%) compared with NR-DLR and in 23 of 40 cases (57.5%) compared with HIR. Diagnostic conversion from a diagnostically insufficient score to a diagnostic score occurred in 3 of 40 cases (7.5%) compared with NR-DLR and in 11 of 40 cases (27.5%) compared with HIR. In addition, SR-DLR received the highest scores for the ease of evaluating tumor–vessel interface for vascular invasion, image noise, and image sharpness. All differences were statistically significant compared with NR-DLR and HIR (all *p* < 0.001). Diagnostic acceptability and tumor conspicuity were also rated highest with SR-DLR, with statistically significant differences relative to both NR-DLR and HIR (all *p* < 0.001). For diagnostic acceptability, no patients received a score of 2 or lower in the SR-DLR, NR-DLR, or HIR. Inter-reader agreement was generally poor for all qualitative assessment items, with ICC(2,1) values ranging from 0.04 to 0.20.

**Table 2:** Results of the quantitative image analyses

	Result, mean ± SD			Comparison, *p* value	
	SR-DLR	NR-DLR	HIR	SR-DLR vs. NR-DLR	SR-DLR vs. HIR
CT_aorta1_ (HU)	299.8 ± 44.6	295.2 ± 43.6	297.5 ± 44.0	< 0.001*	< 0.001*
CT_aorta2_ (HU)	225.0 ± 66.0	220.4 ± 65.8	222.1 ± 66.2	< 0.001*	< 0.001*
CT_pancreas_ (HU)	123.2 ± 24.5	119.1 ± 24.1	120.2 ± 24.2	< 0.001*	< 0.001*
Noise_1_ (HU)	9.23 ± 1.1	10.7 ± 1.9	18.2 ± 3.6	< 0.001*	< 0.001*
Noise_2_ (HU)	9.18 ± 1.0	10.9 ± 1.9	18.3 ± 3.5	< 0.001*	< 0.001*
CNR	11.12 ± 7.63	9.32 ± 6.25	5.66 ± 4.01	< 0.001*	< 0.001*
ERSartery (HU/mm)	158.7 ± 68.7	131.5 ± 45.8	112.3 ± 51.4	0.002*	< 0.001*
ERS_pancreas_ (HU/mm)	156.1 ± 50.0	124.3 ± 40.7	109.6 ± 41.5	< 0.001*	< 0.001*
ERDartery (mm)	0.79 ± 0.34	0.92 ± 0.29	1.12 ± 0.43	< 0.001*	< 0.001*
ERD_pancreas_ (mm)	0.61 ± 0.18	0.73 ± 0.24	0.95 ± 0.55	0.001*	< 0.001*

Comparisons were conducted using paired *t* tests

*SR-DLR* super-resolution deep learning reconstruction, *NR-DLR* normal-resolution deep learning reconstruction, *HIR* hybrid iterative reconstruction, *CT*_*aorta1*_ CT attenuation of the aorta on early arterial phase, *HU* Hounsfield unit, *CT*_*aorta2*_ CT attenuation of the aorta on late arterial phase, *CT*_*pancreas*_ CT attenuation of the pancreatic parenchyma on late arterial phase, *Noise*_*1*_ Noise on early arterial phase, *Noise*_*2*_ Noise on late arterial phase, *CNR* contrast-to-noise ratio, *ERS* edge rise slope, *ERS*_*artery*_ ERS of the superior mesenteric artery, *ERS*_*pancreas*_ ERS of the pancreatic parenchyma, *ERD* edge rise distance, *ERD*_*artery*_ ERD of the superior mesenteric artery, ERD_pancreas_ ERD of the pancreatic parenchyma

*Statistically significant difference (*p* < 0.025 [= 0.050/2])

**Table 3:** Results of the qualitative image analyses

	Result, median (interquartile range) [mean]			Comparison, *p* value		ICC (2,1) [95% CI]
	SR-DLR	NR-DLR	HIR	SR-DLR vs. NR-DLR	SR-DLR vs. HIR	
Depiction of major peripancreatic arteries						
Celiac artery	4.0 (4.0–4.0) [4.00]	4.0 (4.0–4.0) [3.77]	4.0 (4.0–4.0) [3.72]	NA	NA	0.04 [0.02–0.06]
Common hepatic artery	4.0 (4.0–4.0) [4.00]	4.0 (4.0–4.0) [3.74]	4.0 (4.0–4.0) [3.70]	NA	NA	0.05 [0.03–0.07]
Proper hepatic artery	4.0 (4.0–4.0) [3.99]	4.0 (4.0–4.0) [3.73]	4.0 (4.0–4.0) [3.64]	NA	NA	0.06 [0.04–0.08]
Superior mesenteric artery	4.0 (4.0–4.0) [4.00]	4.0 (4.0–4.0) [3.77]	4.0 (4.0–4.0) [3.70]	NA	NA	0.04 [0.02–0.06]
Splenic artery	4.0 (4.0–4.0) [3.98]	4.0 (4.0–4.0) [3.73]	4.0 (4.0–4.0) [3.65]	NA	NA	0.06 [0.04–0.09]
Gastroduodenal artery	4.0 (4.0–4.0) [3.96]	4.0 (4.0–4.0) [3.68]	4.0 (4.0–4.0) [3.58]	NA	NA	0.07 [0.05–0.10]
Depiction of small peripancreatic arteries						
Pancreaticoduodenal arcade system	4.0 (4.0–4.0) [3.76]	3.0 (3.0–4.0) [3.29]	3.0 (3.0–3.0) [3.13]	< 0.001*	< 0.001*	0.11 [0.06–0.16]
Dorsal pancreatic artery–transverse pancreatic artery–great pancreatic artery system	3.0 (3.0–4.0) [3.40]	3.0 (3.0–3.0) [2.87]	3.0 (2.0–3.0) [2.71]	< 0.001*	< 0.001*	0.11 [0.05–0.16]
The ease of evaluating tumor–vessel interface for vascular invasion	4.0 (4.0–4.0) [3.74]	3.5 (3.0–4.0) [3.40]	3.0 (3.0–3.0) [3.06]	< 0.001*	< 0.001*	0.11 [0.05–0.16]
Image noise	4.0 (4.0–4.0) [3.60]	3.0 (3.0–3.0) [3.22]	2.0 (2.0–3.0) [2.68]	< 0.001*	< 0.001*	0.20 [0.12–0.29]
Image sharpness	4.0 (4.0–4.0) [3.63]	3.0 (3.0-3.25) [3.20]	3.0 (2.0–3.0) [2.77]	< 0.001*	< 0.001*	0.16 [0.09–0.23]
Diagnostic acceptability	5.0 (4.0–5.0) [4.36]	4.0 (4.0–4.0) [3.93]	3.0 (3.0–4.0) [3.44]	< 0.001*	< 0.001*	0.20 [0.11–0.29]
Tumor conspicuity	4.0 (4.0–4.0) [3.51]	3.0 (3.0–3.0) [3.16]	3.0 (3.0–3.0) [2.79]	< 0.001*	< 0.001*	0.17 [0.10–0.25]

Data are presented as median (interquartile range), with mean values of the five readers provided for reference

Comparisons were conducted using Wilcoxon signed-rank test

*SR-DLR* super-resolution deep learning reconstruction, *NR-DLR* normal-resolution deep learning reconstruction, *HIR* hybrid iterative reconstruction, *ICC* intraclass correlation coefficient

*Statistically significant difference (*p* < 0.025 [= 0.050/2])

## Discussion

This study compared three reconstruction algorithms—SR-DLR, NR-DLR, and HIR—with respect to the visualization of peripancreatic arteries and diagnostic acceptability in patients with pancreatic cancer. SR-DLR showed significantly better quantitative image quality than both NR-DLR and HIR, including lower image noise, higher CNR, higher ERS, and lower ERD. In the qualitative analyses, significant improvements were observed with SR-DLR for the depiction of small peripancreatic arteries, the tumor–vessel interface, image noise, image sharpness, diagnostic acceptability, and tumor conspicuity. In contrast, no significant differences were observed for the depiction of major peripancreatic arteries.

In recent years, DLR has emerged as a transformative technique capable of overcoming the inherent limitations of conventional HIR [14, 16]. Nagayama et al. reported that SR-DLR (PIQE) with 1024-matrix reconstruction achieved lower image noise, higher CNR, and improved tumor conspicuity for PDAC compared with NR-DLR and HIR [18]. Our findings are consistent with these results and further extend them to the evaluation of the small peripancreatic arteries in PDAC. Similarly, Nakamoto et al. reported that SR-DLR enables both noise reduction and improved CNR in contrast-enhanced abdominal CT, resulting in improved visualization of fine structures [17].

In this study, CT attenuation of both the aorta and pancreatic parenchyma was higher with SR-DLR than with NR-DLR and HIR. Iterative reconstruction and deep learning–based reconstruction algorithms are nonlinear processes and may produce small systematic shifts in mean attenuation values, as reported in previous studies of SR-DLR [17, 18, 20]. However, the absolute differences in aortic attenuation were small, approximately 2–5 HU among the three reconstruction methods. Therefore, although the differences in CT attenuation of the aorta reached statistical significance, their magnitude was unlikely to be clinically meaningful.

Arteries supplying the pancreas are inherently small in caliber, and their precise evaluation requires high spatial resolution. SR-DLR, trained on ultra-high-resolution CT data, restores fine vascular structures from standard detector data while suppressing noise, thereby overcoming the traditional trade-off between spatial resolution and image noise [20]. In the present study, SR-DLR was reconstructed with a 1024 × 1024 matrix, whereas NR-DLR and HIR were reconstructed with a 512 × 512 matrix, reflecting the maximum available matrix size for each method. Therefore, the superior ERS and ERD values may reflect the combined effect of the SR-DLR algorithm and the higher matrix size, rather than the algorithm alone.

In the qualitative analysis, SR-DLR yielded higher scores for smaller peripancreatic arteries, including the pancreaticoduodenal arcade system and the dorsal pancreatic artery–transverse pancreatic artery–great pancreatic artery system, as well as for the tumor–vessel interface. By contrast, no significant differences were observed for major arteries. This was likely because these large arteries were already clearly depicted with NR-DLR and HIR. This ceiling effect may explain why the added value of SR-DLR was more apparent for small-caliber vessels and subtle anatomical interfaces than for major arteries.

In addition, the analysis of score improvement and diagnostic conversion showed that SR-DLR more often improved depiction within the diagnostic range than converted diagnostically insufficient images into diagnostic ones. Therefore, these findings should be interpreted as improved vascular clarity rather than direct evidence of improved diagnostic performance. Inter-reader agreement was generally poor across the qualitative assessment items. This finding suggests that visual assessment of small peripancreatic arteries and the tumor–vessel interface varied somewhat among the readers.

From a clinical perspective, clearer depiction of small peripancreatic arterial systems and the tumor–vessel interface may be useful for preoperative image interpretation in PDAC. Although current resectability criteria primarily focus on major arteries such as the celiac artery and superior mesenteric artery [21], detailed visualization of smaller arterial branches, anatomical variations, and collateral pathways may provide additional information for safer and more individualized surgical planning [22]. There are some limitations with this study. First, it was a retrospective, single-center study with a relatively small sample size (*n* = 40). Larger multicenter prospective studies are needed to validate these findings. Second, SR-DLR is a vendor-specific algorithm, and direct comparisons with deep-learning technologies from other manufacturers were not performed. Third, we focused primarily on the arterial phase to optimize arterial evaluation; the utility of SR-DLR in other phases, such as the portal venous phase for venous invasion, remains to be fully elucidated. Fourth, the study cohort included a relatively small number of patients, and subgroup analyses regarding the effect of tumor-, patient-, and scan-related factors on vessel depiction and the tumor-vessel interface was not conducted. Further investigations in larger cohorts are warranted. Fifth, this study evaluated image quality, vascular depiction, tumor–vessel interface visualization, and diagnostic acceptability, rather than diagnostic performance or clinical outcomes. Because this study did not evaluate diagnostic accuracy for arterial invasion, resectability classification, surgical findings, or treatment decision-making, the clinical impact of improved vascular clarity remains uncertain and should be clarified in future studies. Finally, ERS and ERD were not measured directly in small peripancreatic arteries. Because these arteries are small in caliber and often have variable or oblique courses, direct placement of linear ROIs perpendicular to the vessel wall is technically challenging and may be susceptible to partial volume effects and limited reproducibility. For this reason, the superior mesenteric artery was used as a reproducible reference artery for quantitative sharpness assessment. Therefore, the quantitative sharpness results should be interpreted as reflecting improved edge sharpness in reference structures, whereas the improved depiction of small peripancreatic arteries was supported by the qualitative reader assessment.

In conclusion, SR-DLR improved quantitative image quality and enhanced qualitative depiction of small peripancreatic arteries and the tumor–vessel interface compared with NR-DLR and HIR in pancreatic dynamic CT for patients with PDAC. These findings suggest that SR-DLR may facilitate preoperative image interpretation in patients with PDAC.

## Data Availability

The datasets generated and/or analyzed during the current study are not publicly available due to patients confidentiality.
